# CD302 regulates the malignant phenotypes of lung adenocarcinoma as a tumor suppressor gene

**DOI:** 10.3389/fonc.2025.1601706

**Published:** 2025-11-14

**Authors:** Kai Wang, Yao Wang, Tao Lu, Manjun Gao, Yongxiang Song, Cheng Chen, Xixian Ke

**Affiliations:** 1 Department of Thoracic Surgery, Affiliated Hospital of Zunyi Medical University, Zunyi, Guizhou, China; 2 Department of Health Management, Affiliated Hospital of Zunyi Medical University, Zunyi, Guizhou, China; 3 The Public Experimental Center of Medicine, Affiliated Hospital of Zunyi Medical University, Zunyi, Guizhou, China

**Keywords:** cluster of differentiation 302 (CD302), lung adenocarcinoma (LUAD), bioinformatics analysis, clinical data analysis, experimental validation

## Abstract

**Background:**

CD302 encodes a transmembrane glycoprotein involved in immune regulation via cell–extracellular matrix interactions. Its role in lung adenocarcinoma (LUAD) remains unexplored. This study investigates CD302’s expression, clinical relevance, and functional mechanisms in LUAD.

**Methods:**

Using public databases and bioinformatics, CD302 expression and clinical significance were analyzed. Validation was performed in 52 paired NSCLC samples from the Department of Thoracic Surgery at the Affiliated Hospital of Zunyi Medical University using RT−qPCR and clinical correlation analysis. *In vitro* CD302 overexpression models in A549 and PC-9 cells were used to assess malignant phenotypes.

**Results:**

Database analysis revealed that CD302 exhibited low expression in lung adenocarcinoma tissues, with its expression levels being negatively correlated with T stage, N stage, and TNM stage. Patients with high CD302 expression demonstrated significantly higher overall survival (OS) and progression-free survival (PFS). Both univariate and multivariate Cox regression analyses identified CD302 expression as an independent prognostic factor for LUAD patients. A nomogram was constructed to predict 1-, 3-, and 5-year survival rates, and calibration curve analyses confirmed the model’s robust predictive capability. The area under the ROC curve (AUC) of 0.912 further suggests that CD302 holds substantial diagnostic potential. Analysis of clinical NSCLC samples validated the low expression of CD302 in lung adenocarcinoma, with expression levels showing a negative correlation with tumor diameter (correlation coefficient = -0.5358). Moreover, using the human lung adenocarcinoma cell lines A549 and PC-9, a CD302 overexpression model was established. Subsequent CCK-8, colony formation, wound healing, and transwell invasion assays demonstrated that CD302 overexpression inhibits the proliferation, migration, and invasion of NSCLC cells.

**Conclusion:**

CD302 is expressed at low levels in lung adenocarcinoma tissues, and its expression is negatively correlated with tumor diameter, serving as an independent risk factor for poor prognosis in lung adenocarcinoma patients. Overexpression of CD302 inhibits the proliferation, migration, and invasion of A549 and PC-9 cells. Therefore, CD302 holds potential as a diagnostic and prognostic biomarker for LUAD patients.

## 1 Introduction

Lung cancer is one of the most common cancers worldwide, accounting for 12.4% of all cancer cases (1) and representing the leading cause of cancer-related death globally (2). In China, lung cancer remains one of the most prevalent malignancies and a primary cause of cancer mortality (3). Lung cancer is mainly classified into non−small cell lung cancer (NSCLC) and small cell lung cancer (SCLC), accounting for approximately 85% and 15% of cases, respectively (4). Due to its insidious onset, early-stage lung cancer is typically asymptomatic, and symptoms generally manifest at advanced stages, resulting in a 5−year survival rate of only about 2.8% (5). Although the emergence of targeted therapies and immunotherapies has improved treatment outcomes for advanced lung cancer patients, issues such as drug resistance and recurrence have hindered significant improvements in survival rates. Consequently, there is a critical need to identify novel biomarkers to facilitate early diagnosis and to provide new therapeutic targets (6–8).

CD302, a member of the C−type lectin family, encodes a transmembrane glycoprotein. It is derived from the 428′ terminal splice variant transcript of the Hodgkin’s lymphoma cell L3 clone gene. As the simplest single−domain type I C−type lectin receptor in this family, CD302 is also known as BIMLE and CDEC−205−related C−type lectin−1 (DCL−1/CLEC13A) (9, 10). CD302 is most highly expressed in the human liver, followed by the lung and spleen, and is predominantly expressed on myeloid cells (such as dendritic cells and macrophages). By modulating dendritic cell migration, cytoskeletal dynamics, and the distribution of immune cells, CD302 plays a crucial role in immune responses and homeostasis, thereby influencing the biological behavior of the tumor microenvironment (11–13). The function of CD302 is closely associated with its carbohydrate recognition domain (CRD), which has an independent folding structure. Through synergistic interaction with calcium ions, this domain efficiently recognizes carbohydrate molecules—including mannose, glucose, galactose, and glycosaminoglycans—that are important in immune responses (14, 15).

In primary acute myeloid leukemia (AML), CD302 is primarily expressed in leukemia stem cells (LSCs) and progenitor cells, with its expression being relatively limited in normal hematopoietic cells. A monoclonal antibody targeting CD302 (MMRI−20) has been shown to induce antibody−dependent cellular cytotoxicity (ADCC) by recruiting effector cells such as natural killer cells or macrophages to kill AML cells (16). Furthermore, in hepatocytes, CD302 is primarily located intracellularly rather than on the cell surface. Consequently, when conjugated with pyrrolobenzodiazepine (PBD) drugs, MMRI-20 effectively kills AML cell lines such as HL-60, while showing no toxicity toward hepatocytes (e.g., HepG2) that also express CD302. This internalization property makes CD302 an ideal target for antibody-drug conjugates (ADCs) (16, 17).

In multiple myeloma (MM), the expression level of CD302 is closely correlated with patient survival. Patients with high CD302 expression have a significantly longer overall survival (OS) than those with low expression. A CD302 gene-based risk scoring model further stratifies patients into high-risk and low-risk groups. The median survival time in the high-risk group was 50.6 months, whereas the median survival had not been reached in the low-risk group. In low-risk patients, high CD302 expression was associated with significant enrichment of genes related to mature marrow plasma cells, suggesting a potential role in maintaining the differentiated state of plasma cells. In contrast, high-risk MM samples with low CD302 expression exhibited gene signatures of undifferentiated plasmablasts and aberrant activation of MYC oncogene targets (18). MYC is a key oncogenic transcription factor that contributes to tumor progression and poor prognosis (19, 20). Thus, low CD302 expression may be closely associated with abnormal activation of the MYC pathway and the development of multiple myeloma.

In lung squamous cell carcinoma (LUSC), CD302 expression is significantly downregulated in tumor tissues and is positively correlated with angiogenesis, suggesting that it may influence LUSC progression by regulating tumor vascularization. Within regulatory networks, a strong positive correlation exists between SECISBP2L and CD302, indicating that SECISBP2L may positively regulate CD302 and affect downstream angiogenic pathways. This further suggests that CD302 likely functions through the “SECISBP2L–CD302–angiogenesis” axis in LUSC. Moreover, external validation using single-cell RNA sequencing (scRNA-seq) and multi-omics databases (e.g., Human Protein Atlas, Oncomine, GEPIA, LinkedOmics) confirmed CD302 expression and its clinical relevance, demonstrating that low expression is associated with poor patient prognosis. These findings indicate that CD302 may serve as a potential target for LUSC treatment and prognostic evaluation (21). However, research on CD302 in other lung cancer subtypes remains limited, and to date, no studies have reported on its role in LUAD. Given the differences in pathogenesis and clinicopathological features among various lung cancer subtypes, investigating the role of CD302 in LUAD may hold significant research and clinical value.

In this study, public databases, bioinformatics analyses, and validation with clinical tissue samples were employed to demonstrate that CD302 is downregulated in LUAD tissues, with its expression negatively correlated with tumor diameter and exhibiting high diagnostic efficacy. Survival curve analyses revealed that patients with high CD302 expression have significantly higher OS and PFS. Furthermore, univariate and multivariate Cox regression analyses identified CD302 expression as an independent prognostic factor for LUAD patients, and a nomogram model was constructed to predict 1−, 3−, and 5−year survival rates. Finally, *in vitro* experiments, including CCK−8, colony formation, wound healing, and transwell invasion assays, demonstrated that overexpression of CD302 inhibits the proliferation, migration, and invasion of NSCLC cells.

## 2 Materials and methods

### 2.1 Reagents and instruments

Major experimental reagents are listed in **Supplementary Table S1**.

### 2.2 Data collection and analysis

To investigate the role of CD302 in lung adenocarcinoma (LUAD), transcriptomic data and corresponding clinical information were obtained from The Cancer Genome Atlas (TCGA). After merging the cart files and metadata, the expression levels of CD302 were compared between cancerous and normal tissues. Furthermore, the relationship between CD302 expression and clinicopathological characteristics was evaluated. Survival curves were generated using the Kaplan–Meier Plotter database (http://kmplot.com/analysis/) to evaluate the association between CD302 expression and the prognosis of LUAD patients. Univariate and multivariate Cox regression analyses were conducted using RStudio software to examine the relationship between CD302 expression and clinical variables, including age, sex, TNM stage, and pathological stage. Based on these analyses, a nomogram model was constructed to predict the 1-, 3-, and 5-year survival probabilities of LUAD patients. In addition, ROC curves were plotted to evaluate the diagnostic value of CD302 for LUAD. Between January 2021 and December 2023, tumor tissues and adjacent normal tissues (located 2 cm from the tumor margin) were collected from 52 NSCLC patients who underwent surgery in the Department of Thoracic Surgery at Zunyi Medical University Hospital. Inclusion criteria were: (1) a pathological diagnosis of NSCLC; (2) age greater than 18 years; and (3) no preoperative chemotherapy, radiotherapy, targeted therapy, or immunotherapy. Exclusion criteria were: (1) a pathological diagnosis other than NSCLC; (2) age less than 18 years; and (3) preoperative treatment with chemotherapy, radiotherapy, targeted therapy, or immunotherapy. All tissue samples were immediately placed in RNA preservation solution after surgical resection and transferred to a –80°C freezer within 30 minutes. This study was approved by the Ethics Committee of Zunyi Medical University (Ethics Approval No.: KLLY-2023-098).

### 2.3 Cell culture

Human normal lung epithelial cells (BEAS-2B) and NSCLC cell lines (H1299, A549, PC-9, H1975, and H460) were obtained from Wuhan Punosai Life Science Technology Co., Ltd. BEAS-2B cells were cultured in DMEM supplemented with 10% fetal bovine serum (FBS), while the NSCLC cell lines were maintained in RPMI-1640 medium containing 10% FBS. All cells were incubated in a humidified atmosphere at 37 °C with 5% CO2.

### 2.4 Lentiviral transfection

The CD302 overexpression lentiviral vector was commissioned from Shanghai Hanyuan Biotechnology Co., Ltd. Detailed information on the lentiviral plasmids is provided in **Supplementary Table S2**, and maps of the empty vector and the constructed vector are shown in **Supplementary Figure S1**. PCR results demonstrated that the melting curve of CD302 in the lentiviral plasmid differed markedly from those of the empty plasmid and 293T cells (**Supplementary Figure S2**). The primers used in the experiment were listed in **Supplementary Table S3**. Quantitative analysis showed that CD302 was highly expressed in the lentiviral plasmid, confirming successful plasmid construction (**Supplementary Table S4**). Recombinant plasmids were transfected into 293T cells for virus packaging; supernatants were collected, concentrated, and used at different dilutions to infect 293T cells (**Supplementary Figure S3**). The mean viral titer was determined to be 4.28 × 10^8 (**Supplementary Table S5**). Using an MOI of 10, A549 and PC-9 cells were infected and selected with puromycin, resulting in stable cell lines that overexpress CD302.

### 2.5 Quantitative real−time PCR

RNA Extraction and Reverse Transcription: RNA from cells and tissues was extracted using TRIzol reagent. Cells were washed with pre-chilled PBS and collected by adding TRIzol, while tissue samples were minced, combined with magnetic beads, and homogenized. Subsequently, chloroform was added, the mixture was vortexed thoroughly, and centrifugation was performed to achieve phase separation. The upper aqueous phase was collected, and an equal volume of pre-chilled isopropanol was added to precipitate the RNA. The RNA pellet was then washed with 75% ethanol, air-dried, dissolved in RNase−free water, and its purity was assessed. Next, reverse transcription was carried out at 37 °C using a reverse transcription kit to synthesize cDNA from the RNA template (**Supplementary Table S6**). Finally, the TB Green PCR system along with specific primers was utilized for amplification, and CD302 expression was quantified using the 2^-ΔΔCT method (**Supplementary Table S7**). Primers used are presented in **Supplementary Table S8**.

### 2.6 Western blot

Cells were lysed using protein lysis buffer, and the resulting mixture was transferred to a 1.5 mL Eppendorf tube. The lysate was centrifuged at 12,000 g for 30 minutes at 4 °C, and the supernatant was collected. Protein concentration was then determined using the BCA assay, and the protein solution was adjusted to 2 µg/µL using PBS. Subsequently, samples were boiled for 10 minutes to ensure complete denaturation prior to storage. For electrophoresis, samples were resolved on a gel composed of a 10% resolving gel and a 5% stacking gel (**Supplementary Table S9**). Following sample loading, electrophoresis was conducted at 80 V for 30 minutes (stacking phase) and 120 V for 90 minutes (resolving phase). After transferring the proteins onto a PVDF membrane via electroblotting, the membrane was blocked at room temperature, incubated overnight at 4°C with primary antibodies, and then incubated with secondary antibodies at room temperature for 1 hour. Finally, images were captured using ECL detection and analyzed with appropriate software. Antibodies used are presented in **Supplementary Table S10**.

### 2.7 CCK-8 assay

Cells from the control and experimental groups in the logarithmic phase were seeded into 96-well plates at a density of 1,000 cells per well, with five replicates per group and an additional blank control. PBS was added to the peripheral wells to prevent evaporation. After allowing the cells to adhere for 6–8 hours, the culture medium was replaced, and a CCK-8 solution was prepared at a 10:1 ratio (medium to CCK-8 reagent) before being added to each well. The plates were then incubated in a cell culture incubator for 30 minutes, after which the absorbance at 450 nm was measured. This procedure was repeated every 24 hours for a total of 3 days, and cell proliferation curves were generated based on the absorbance values obtained at each time point.

### 2.8 Colony formation assay

After digestion and centrifugation, cells from the control and experimental groups were resuspended and counted. The single-cell suspension was diluted to a concentration of 500 cells/mL, and 1 mL of the suspension was seeded into each well of a 6−well plate, with three replicates per group. The cells were cultured at 37°C in a humidified atmosphere containing 5% CO_2_ for 1–2 weeks, with observations every 3–4 days. Once distinct colonies were observed, the culture was terminated. The medium was discarded and the cells were washed three times with PBS, then fixed with 1 mL of 4% paraformaldehyde for 30 minutes. After fixation, the cells were washed three times with PBS, stained for 15 minutes, and then washed and air-dried. Finally, images were captured, and the number of colonies was quantified using ImageJ software to assess cell proliferation.

### 2.9 Scratch assay

Cells from the experimental and control groups, exhibiting healthy growth, were digested, centrifuged, resuspended, and counted before being seeded into 6−well plates. They were cultured for 24 hours until reaching 90% confluence. Once confluence was achieved, a scratch was made vertically using a 200 μL pipette tip, and PBS was used to wash away any detached cells. Finally, cell images were captured using an inverted microscope at 0 and 24 hours post-scratch, and the migration rates for each group were recorded and calculated.

### 2.10 Transwell invasion assay

The serum-free medium and matrigel were mixed at an 8:1 ratio at 4 °C. Subsequently, 60 μL of the mixture was added to each upper chamber and incubated for 3 hours to form a thin gel layer. Excess liquid was aspirated, and 100 μL of serum-free medium was added to each chamber, followed by incubation for 30 minutes in a humidified incubator to hydrate the basement membrane. Log-phase cells were prepared as a single-cell suspension and adjusted to a density of 2×10^2^ cells/mL. The lower chamber was filled with 700 μL of complete medium, while 200 μL of the cell suspension was added to the upper chamber. After 24 hours of co-culture, the medium in the upper chamber was discarded, and non-invaded cells were gently removed using a cotton swab. The chambers were washed with PBS, fixed with 4% paraformaldehyde for 30 minutes, and stained with 0.1% crystal violet solution for 10 minutes. After rinsing, invaded cells were photographed under a microscope and quantified.

### 2.11 Statistical analysis

All experimental data were analyzed using SPSS 29.0 statistical software. Normality of the data distribution was assessed via the Shapiro-Wilk test. For normally distributed data, differences between two groups were evaluated using Student’s t-test, while one-way ANOVA was applied for multi-group comparisons. For skewed distributions, nonparametric Mann-Whitney U tests were utilized to assess intergroup differences. Categorical variables in 2×2 contingency tables were analyzed using Fisher’s exact chi-square test. A two-tailed P < 0.05 was considered statistically significant. Graphical representations were generated using GraphPad Prism 8.0 and R 4.3 software.

## 3 Results

### 3.1 CD302 exhibits differential expression in lung adenocarcinoma

**Figure 1:**
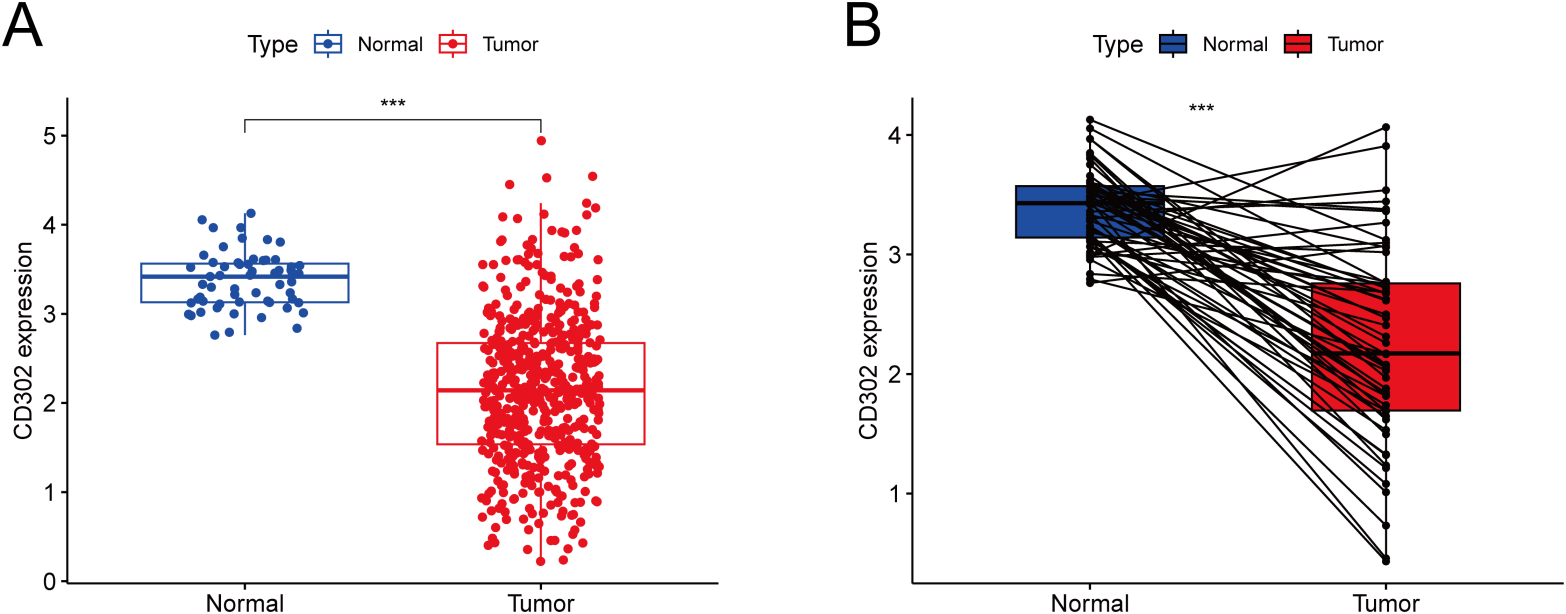
Differential expression of CD302 in LUAD tissues from the TCGA database. **(A)** CD302 expression in unpaired tissues. **(B)** CD302 expression in paired tissues. ***P<0.001.

Gene expression profiling of 598 LUAD samples (59 normal tissues and 539 tumor tissues) from The Cancer Genome Atlas (TCGA) database demonstrated that CD302 expression was significantly downregulated in both unpaired and paired LUAD tissues compared to normal controls (both P < 0.001) (**Figure 1**).

### 3.2 CD302 expression correlates with clinicopathological characteristics in lung adenocarcinoma patients

Using the TCGA database, correlation analyses were conducted between CD302 expression levels and clinical characteristics, including age, gender, T stage, N stage, M stage, and TNM stage.

**Figure 2:**
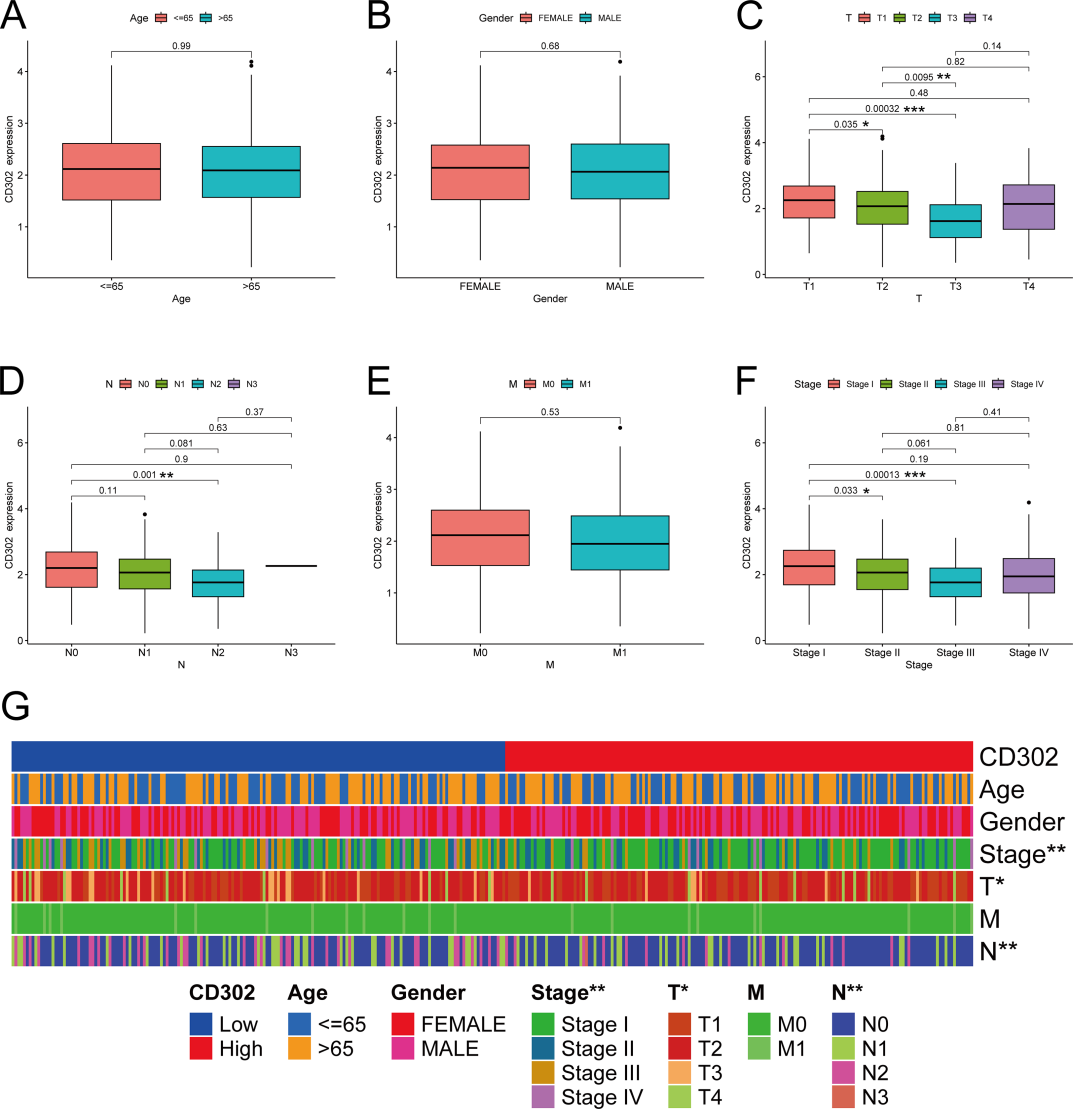
CD302 was differentially expressed in age **(A)**, gender **(B)**, T stage **(C)**, N stage **(D)**, M stage **(E)** and stage **(F)**. **(G)** heatmap of CD302 expression in LUAD tissues based on clinicopathological features. *P<0.05, **P<0.01, and ***P<0.001.

For the correlation between CD302 expression and age, the 539 patients were divided into two groups based on the median age of the cohort: ≤65 years and >65 years. Age grouping served as the independent variable (a dichotomous variable), while CD302 expression level was the dependent variable (a continuous variable). An independent samples t-test was used for statistical analysis, which revealed no significant correlation between CD302 expression and age (P = 0.99) (**Figure 2A**).

For the correlation with gender, patients were categorized into Female and Male groups. Gender was treated as the independent variable (a dichotomous variable), and CD302 expression as the dependent variable (a continuous variable). An independent samples t-test was applied, showing no significant association between CD302 expression and gender (P = 0.68) (**Figure 2B**).

In the analysis of CD302 expression and T stage, patients were stratified into four groups: T1, T2, T3, and T4. T stage was considered an ordered categorical independent variable with four levels, and CD302 expression remained a continuous dependent variable. The Kruskal-Wallis test (a non-parametric rank-sum test) was used for analysis. The results indicated a significant association between CD302 expression and T stage (P < 0.05). Specifically, CD302 expression was higher in T1 than in T2 (P < 0.05), higher in T2 than in T3 (P < 0.01), and higher in T1 than in T3 (P < 0.001), suggesting that CD302 expression decreased with higher T stage (**Figure 2C**).

For the correlation with N stage, patients were classified into four groups: N0, N1, N2, and N3. N stage was treated as an ordered categorical independent variable, and CD302 expression as a continuous dependent variable. The Kruskal-Wallis test was employed, revealing a significant correlation (P < 0.01). CD302 expression was higher in N0 than in N2 (P < 0.01), indicating that expression decreased with higher N stage (**Figure 2D**).

Regarding M stage, patients were divided into M0 and M1 groups. M stage was considered an ordered dichotomous independent variable, and CD302 expression a continuous dependent variable. The Mann-Whitney U test (a non-parametric rank-sum test) was used, and no significant association was found (P = 0.53) (**Figure 2E**).

For TNM stage, patients were grouped into Stage I, Stage II, Stage III, and Stage IV. TNM stage was treated as an ordered categorical independent variable, and CD302 expression as a continuous dependent variable. The Kruskal-Wallis test demonstrated a significant association (P < 0.01). CD302 expression was higher in Stage I than in Stage II (P < 0.05) and higher in Stage I than in Stage III (P < 0.001), indicating that expression decreased with higher TNM stage (**Figure 2F**).

In summary, CD302 expression levels were not associated with age, gender, or M stage, but were significantly correlated with T stage, N stage, and TNM stage (**Figure 2G**). Moreover, CD302 expression progressively decreased with higher disease stage, and these trends were statistically significant.

### 3.3 Prognostic, predictive, and diagnostic value of CD302 expression in lung adenocarcinoma

**Figure 3:**
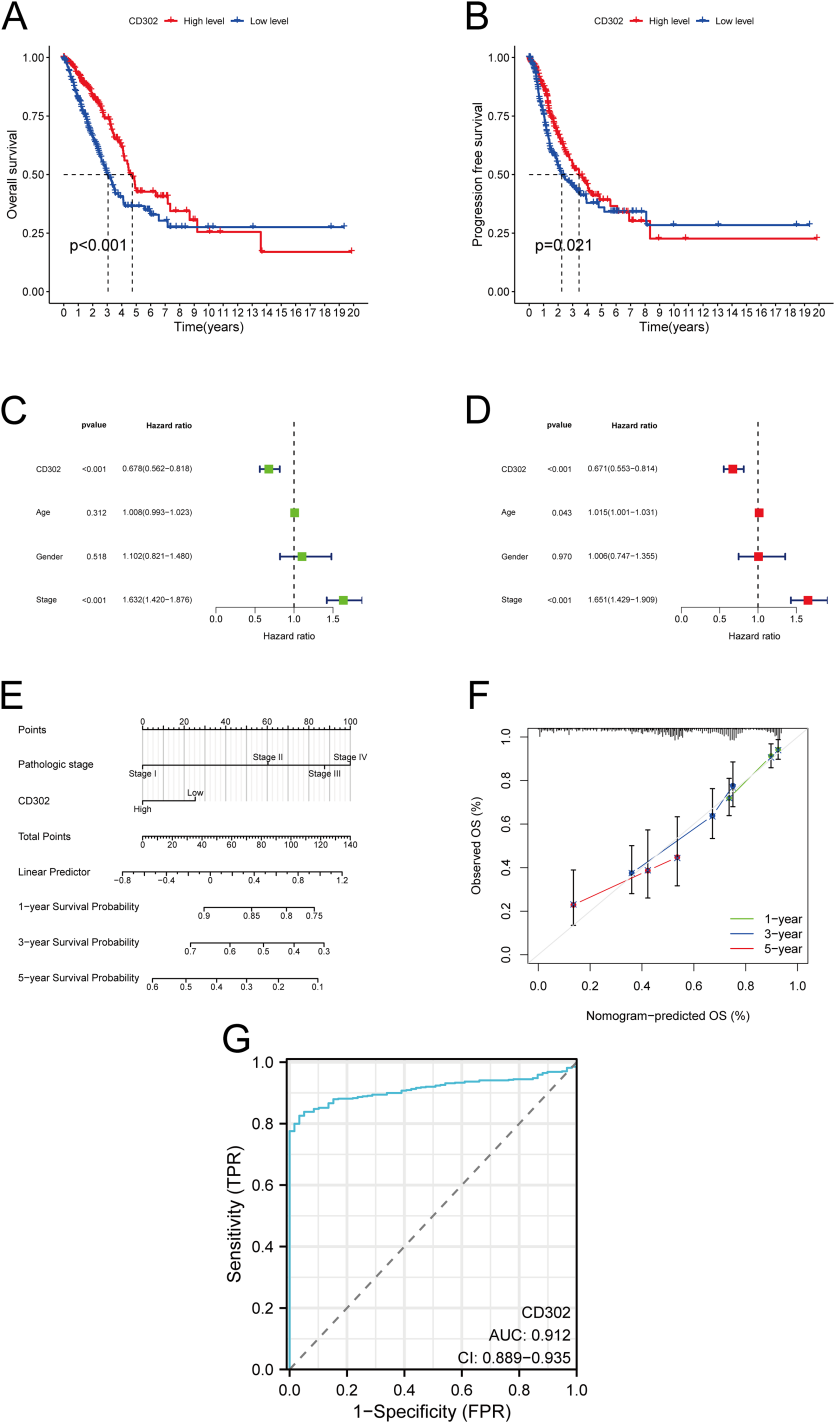
Prognostic, predictive, and diagnostic significance of CD302 expression in lung adenocarcinoma. **(A, B)** Association between CD302 expression levels and OS or PFS in LUAD patients. **(C, D)** Univariate and multivariate Cox regression analyses. **(E, F)** Nomogram model for predicting 1-, 3-, and 5-year survival probabilities and corresponding calibration curves assessing prediction accuracy. **(G)** ROC curve analysis evaluating the diagnostic efficacy of CD302 in LUAD. *P<0.05 and ***P<0.001.

The association between CD302 expression and prognosis in LUAD patients was analyzed using the Kaplan-Meier Plotter database, with survival curves generated to evaluate outcomes. Results indicated that patients with high CD302 expression exhibited significantly prolonged OS and PFS compared to those with low expression (**Figures 3A, B**). Univariate and multivariate Cox regression analyses, performed via RStudio, further assessed the impact of CD302 expression, age, sex, and TNM stage on LUAD prognosis. Both analyses confirmed CD302 expression as an independent prognostic factor (both P < 0.001) (**Figures 3C, D**). A nomogram model was developed to predict 1-, 3-, and 5-year survival probabilities. Calibration curve analysis demonstrated strong concordance between predicted and observed survival rates, validating the model’s predictive accuracy (**Figures 3E, F**). ROC curve analysis was conducted to evaluate the diagnostic efficacy of CD302, revealing an area under the curve (AUC) of 0.912 (P < 0.001; 95% CI: 0.889–0.935), which underscores its high discriminative power in distinguishing tumor tissues from adjacent normal tissues (**Figure 3G**).

### 3.4 Expression of CD302 in clinical NSCLC tissues and analysis of its clinical characteristics

**Figure 4:**
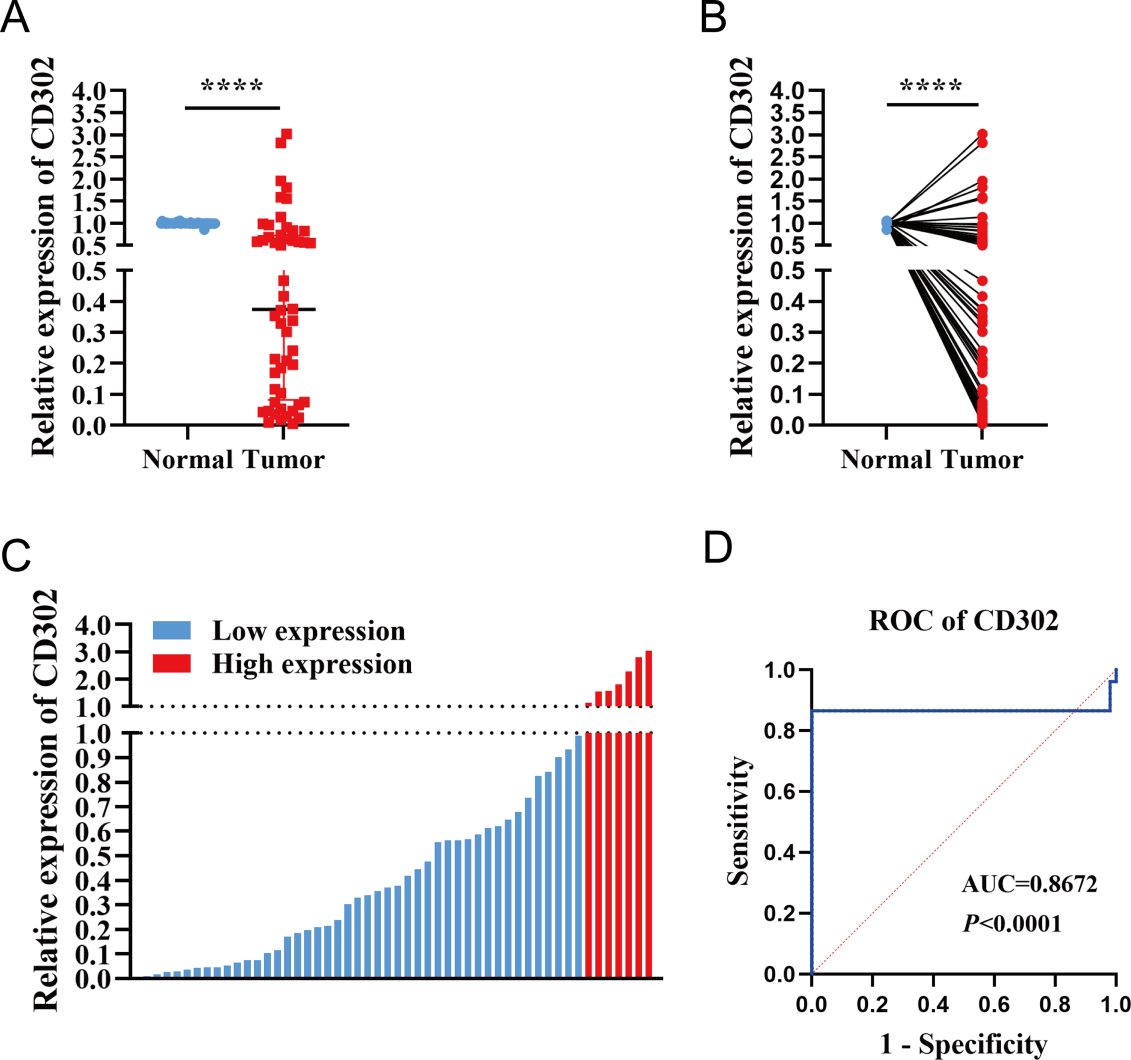
Differential expression of CD302 in clinical NSCLC tissues. **(A)** CD302 expression in unpaired NSCLC tissues. **(B)** CD302 expression in paired NSCLC tissues. **(C)** CD302 expression levels across 52 NSCLC tissue samples. **(D)** Receiver operating characteristic (ROC) curve evaluating the diagnostic efficacy of CD302 in distinguishing NSCLC patients. ****P < 0.0001.

A total of 52 paired NSCLC samples were included in this study. The cohort comprised 29 males and 23 females, aged 20–76 years (mean age: 58 years), with 27 patients aged ≤58 years and 25 patients aged >58 years. TNM staging classified 41 cases as stage I, 6 as stage II, and 5 as stage III. Tumor differentiation grades included 14 well-differentiated, 23 moderately differentiated, and 15 poorly differentiated cases. Pathological subtypes consisted of 42 lung adenocarcinomas and 10 squamous cell carcinomas (**Supplementary Table S11**). RT-qPCR analysis of CD302 expression in 52 NSCLC tissues and paired adjacent normal tissues revealed that CD302 levels were significantly lower in tumor tissues compared to adjacent normal tissues, with 45 cases showing downregulation and only 7 cases exhibiting upregulation in both unpaired and paired comparisons (**Figures 4A-C**). Receiver operating characteristic (ROC) curve analysis demonstrated a diagnostic AUC of 0.8672 (P < 0.0001), indicating strong discriminative potential of CD302 expression in tissue samples (**Figure 4D**).

Using the median CD302 expression as a cutoff, the 52 patients were divided into high- and low-expression groups (n = 26 each) to examine associations with clinicopathological parameters. Chi-square tests showed no significant association between CD302 expression level and age (P = 0.405) or sex (P = 0.402). Rank-sum tests indicated no significant relationships between CD302 expression level and T stage (P = 0.097), N stage (P = 0.409), overall TNM stage (P = 0.114), or differentiation (P = 0.357), whereas a significant difference was observed for tumor diameter (P = 0.043). All cases were M0, so M stage could not be assessed statistically. Thus, CD302 expression level was unrelated to age, sex, T stage, N stage, M stage, TNM stage, and differentiation, but was associated with tumor diameter (**Table 1**).

**Table 1:** CD302 expression in relation to clinicopathological features.

Clinicopathological features	CD302		P value
	Low expression(n=26)	High expression(n=26)	
Age			0.405
<58 years	12	15	
≥5a years	14	11	
Gender			0.402
Male	16	13	
Female	10	13	
T-categories			0.097
T1	12	18	
T2	12	7	
T3-T4	2	1	
N-categories			0.409
N0	22	24	
N1	1	0	
N2-N3	3	2	
M-categories			N/A
M0	26	26	
M1	0	0	
TNM categories			0.114
I	18	23	
II	5	1	
III-IV	3	2	
Differentiation			0.357
Poor	9	6	
Middle	11	12	
Well	6	8	
Tumor diameter			0.041 *
diameter iatcm	1	1	
1cm<diameter iatcm	6	15	
2cm<diameter iatcm	5	6	
3cm<diameter ≤ 4cm	10	4	
4cm<diameter ≤ 4cm	3	0	
diameter>5cm	1	0	

*P < 0.05.

**Figure 5:**
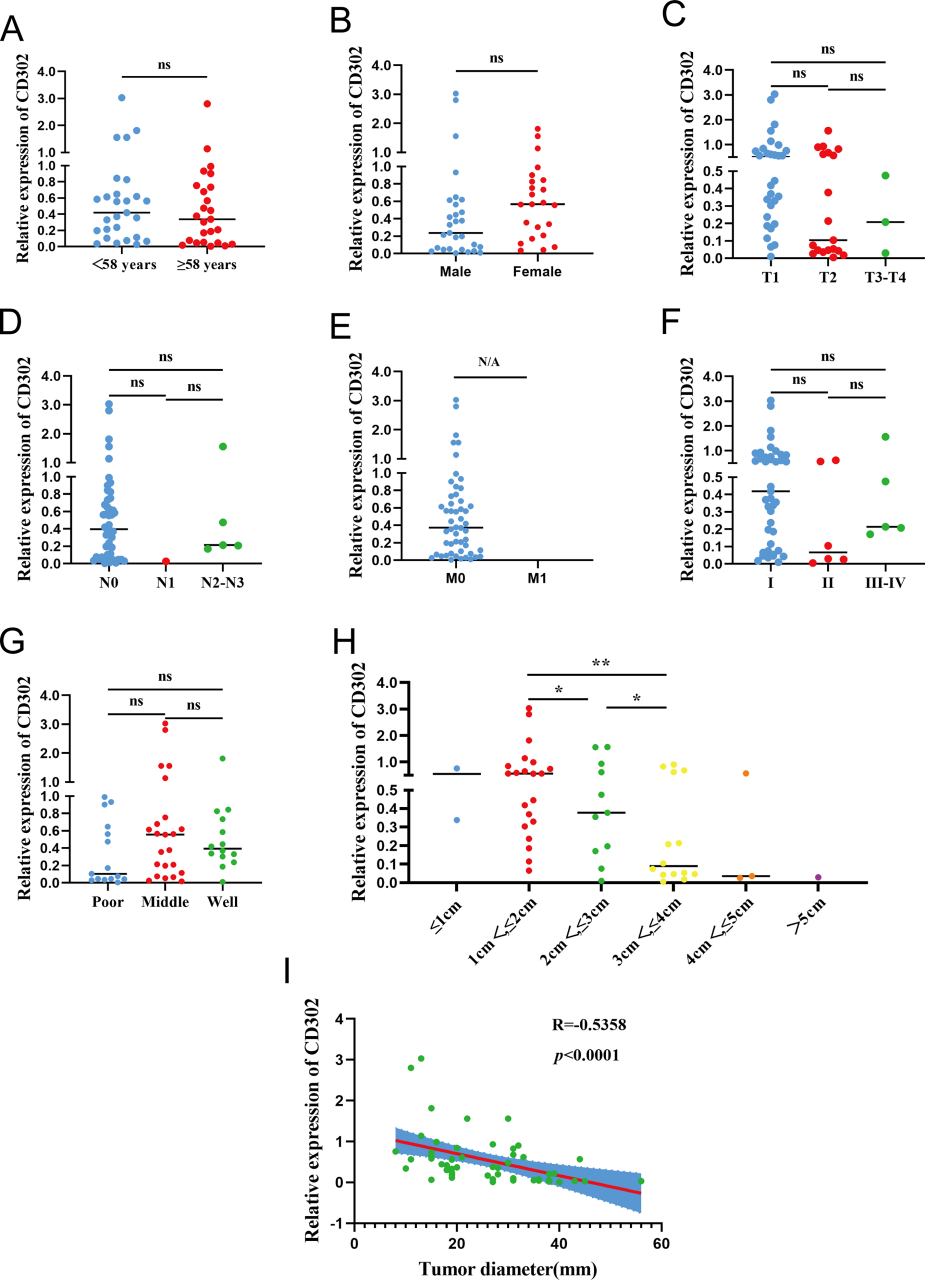
CD302 Expression and clinicopathological correlation in clinical NSCLC tissues. CD302 was differentially expressed in age **(A)**, gender **(B)**, T stage **(C)**, N stage **(D)**, M stage **(E)**, stage **(F)**, differentiation **(G)** and tumor diameter **(H)**. **(I)** The correlation between tumor diameter and CD302 was analyzed using Spearman correlation analysis. ns = not significant, *P<0.05 and **P<0.01. N/A, not applicable.

Correlation analysis between CD302 expression and clinicopathological characteristics in the 52 patients showed no significant associations with age (P = 0.5229), gender (P = 0.5217), T stage (P = 0.1605), N stage (P = 0.7092), TNM stage (P = 0.4074), or differentiation (P = 0.2288). M stage was not tested due to lack of variation. CD302 expression was significantly correlated with tumor diameter (P < 0.05), with expression gradually decreasing as tumor diameter increased. Spearman correlation analysis further confirmed a negative correlation between tumor diameter and CD302 expression (r = -0.5358). Thus, CD302 expression was not associated with age, gender, T stage, N stage, M stage, TNM stage, or differentiation, but was significantly correlated with tumor diameter, with expression decreasing progressively as tumor diameter increased (**Figure 5**).

### 3.5 Expression of CD302 in clinical NSCLC cells and construction and validation of an overexpression cell model

**Figure 6:**
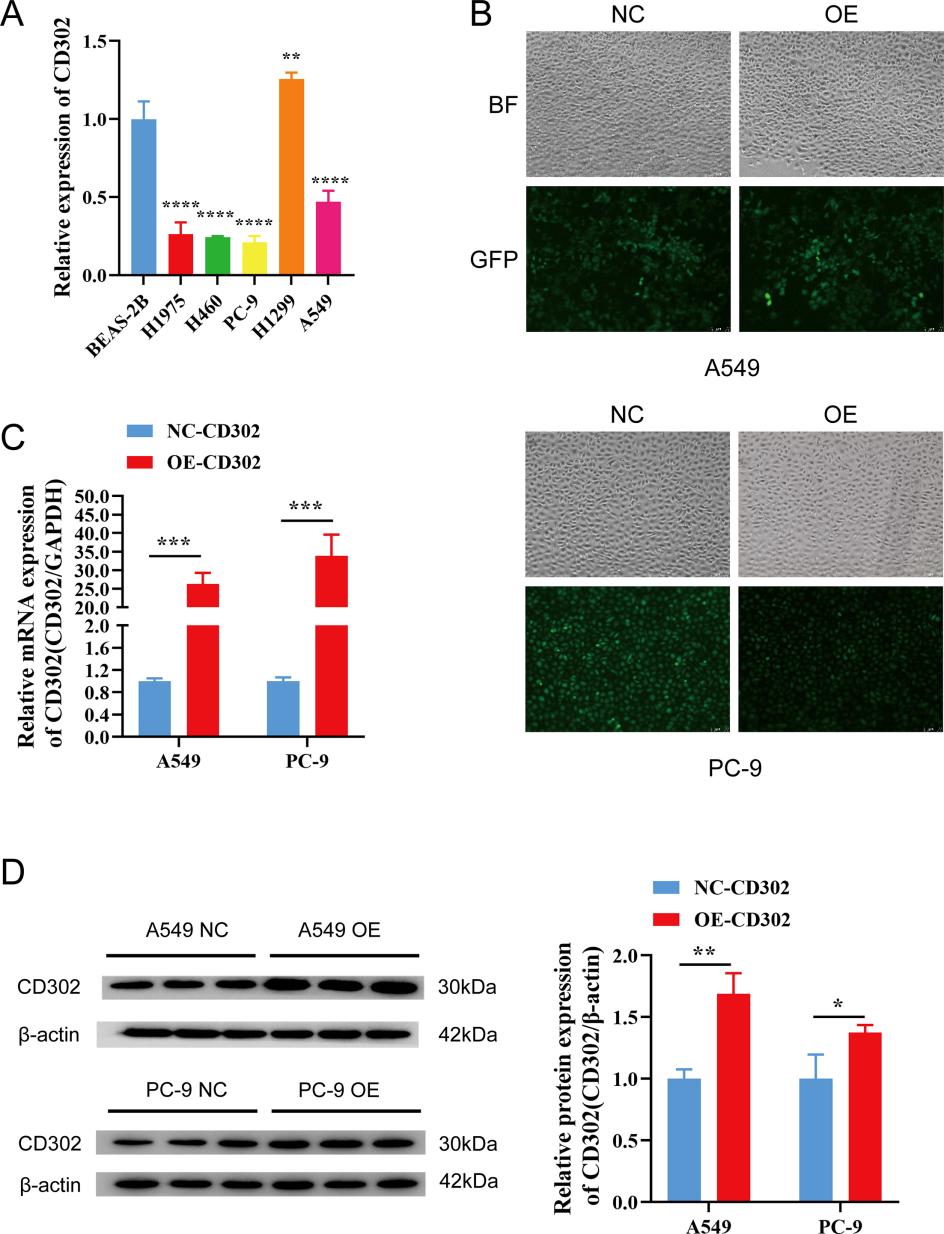
Expression of CD302 in NSCLC cells and construction of the overexpression cell model. **(A)** Expression of CD302 in normal lung epithelial cells and NSCLC cells. **(B)** Transfection efficiency of lentiviral infection in A549 and PC-9 cells observed under a fluorescence microscope. **(C, D)** Expression of CD302 in transfected cells detected by qRT-PCR and Western blot. *P<0.05, **P<0.01, ***P<0.001, and ****P<0.0001.

The expression of CD302 in normal lung epithelial cells (BEAS-2B) and five NSCLC cell lines (H1975, H460, PC-9, H1299, A549) was detected by RT-qPCR. The results showed that CD302 expression was decreased in H1975, H460, PC-9, and A549 cells compared to BEAS-2B cells, whereas it was upregulated in H1299 cells (**Figure 6A**). Considering that H460 is a large-cell carcinoma cell line and H1975 is primarily used for drug resistance studies, A549 and PC-9 cells were selected for the construction of the CD302-overexpressing cell model. Lentiviral particle infection followed by puromycin selection was performed to establish stable CD302-overexpressing A549 and PC-9 cell lines. Fluorescence microscopy revealed an infection efficiency of over 80% (**Figure 6B**). RT-qPCR and Western blot analyses were conducted to assess the mRNA and protein levels of CD302 in control and overexpression groups. The results demonstrated that CD302 expression was significantly upregulated in A549 and PC-9 cells in the overexpression group compared to the control group (**Figures 6C, D**), indicating the successful establishment of the CD302-overexpressing NSCLC cell model.

### 3.6 Effects of CD302 on NSCLC cell proliferation, migration, and invasion

**Figure 7:**
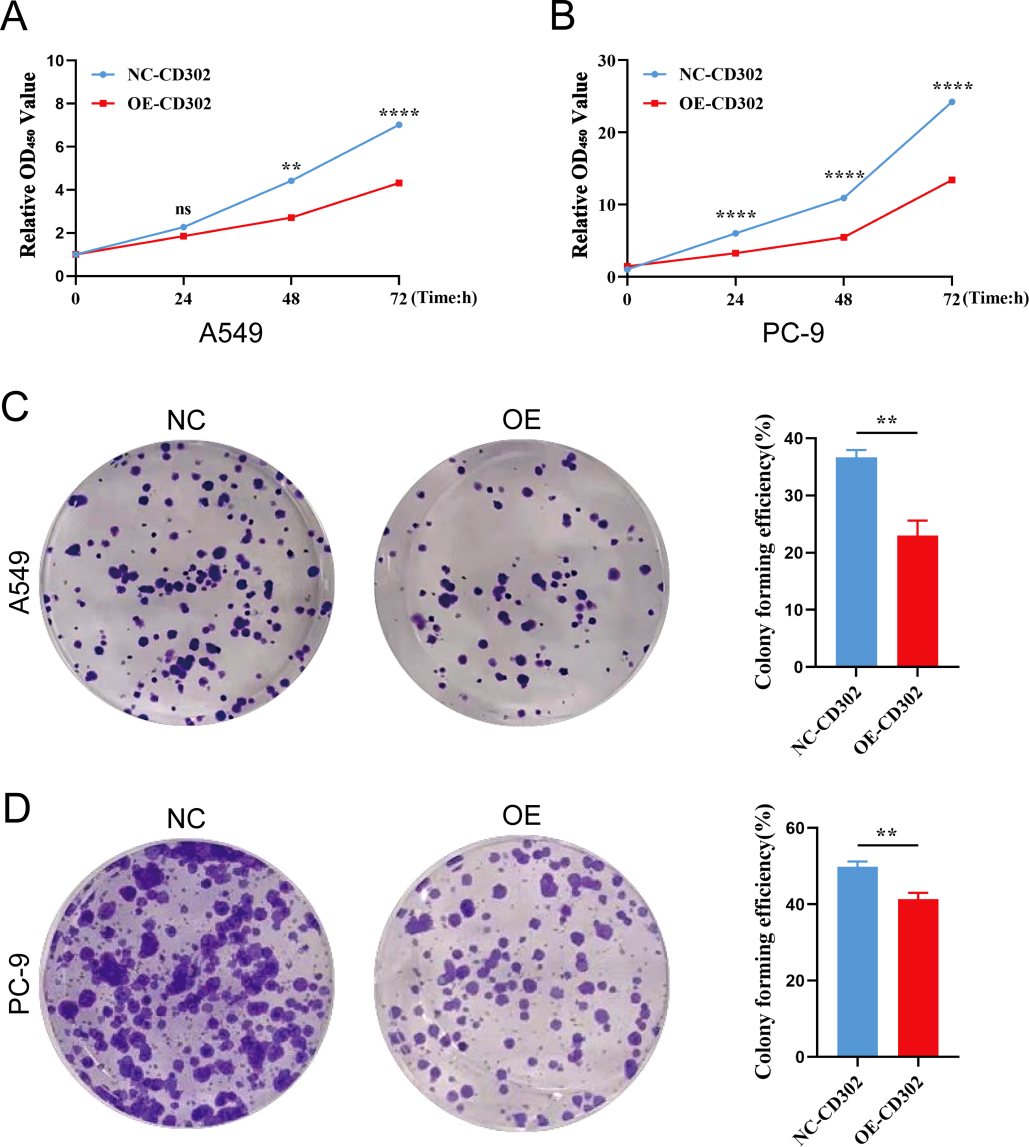
Effects of CD302 on NSCLC cell proliferation. **(A, B)** CCK-8 assay evaluating the effect of CD302 on the proliferation viability of A549 and PC-9 cells. **(C, D)** Colony formation assay assessing the impact of CD302 on the colony-forming ability of A549 and PC-9 cells. **P<0.01 and ****P<0.0001.

**Figure 8:**
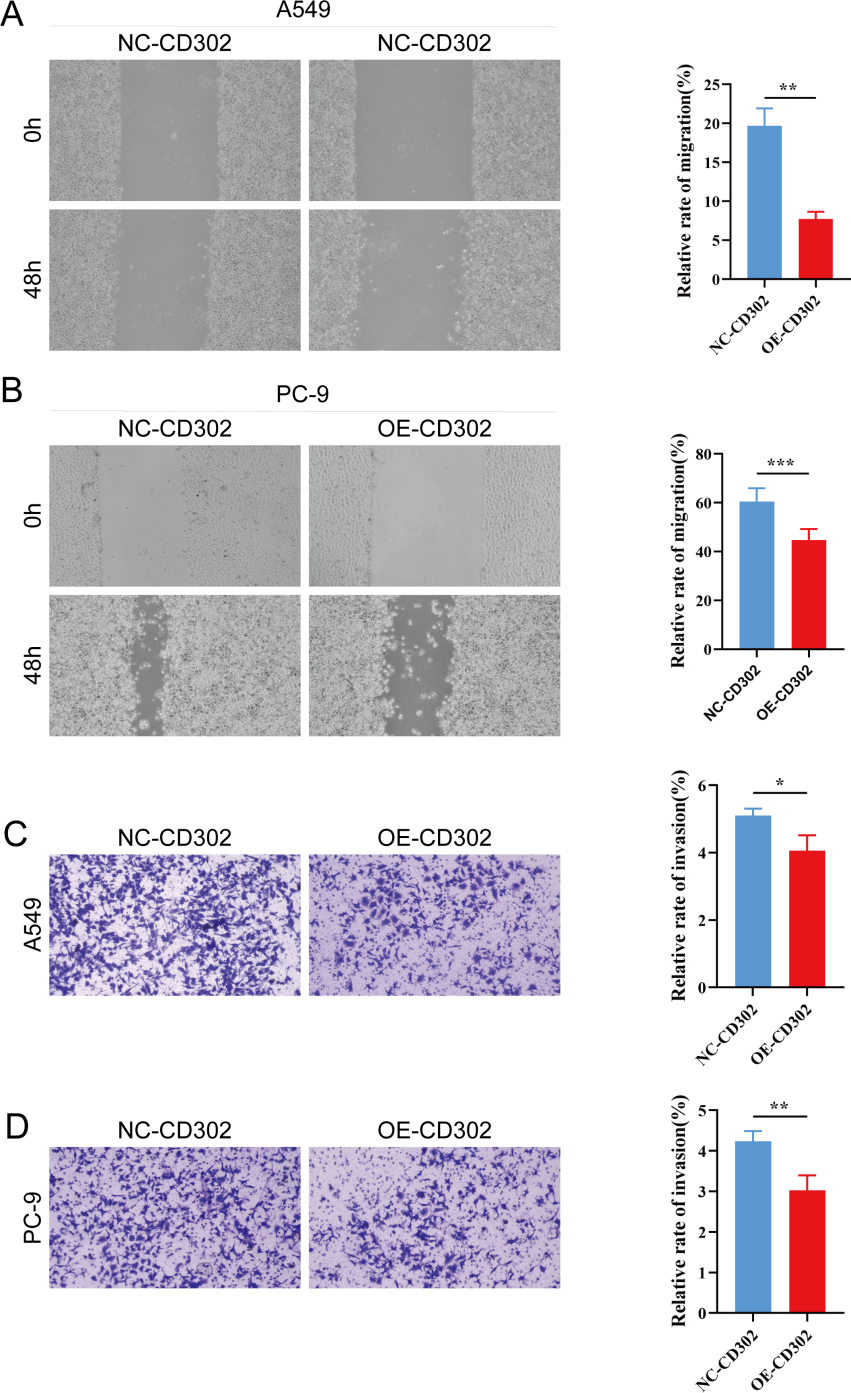
Effects of CD302 on NSCLC cell migration and invasion. **(A, B)** Scratch wound assay evaluating the effect of CD302 on the migration of A549 and PC-9 cells. **(C, D)** Transwell assay assessing the impact of CD302 on the invasion of A549 and PC-9 cells. *P<0.05, **P<0.01 and ***P<0.001.

The CCK-8 assay results showed that cell proliferation viability was reduced in the OE-CD302 group compared to the control group in A549 and H1299 cells (**Figures 7A, B**). The colony formation assay demonstrated that the colony-forming ability of the OE-CD302 group was lower than that of the control group (**Figures 7C, D**). The scratch wound assay indicated that the migration ability of the OE-CD302 group was decreased compared to the control group (**Figures 8A, B**). The Transwell invasion assay results revealed that the invasive capacity of the OE-CD302 group was lower than that of the control group (**Figures 8C, D**).

## 4 Discussion

The C-type lectin family represents one of the most abundant and structurally diverse lectin families found in animals. Its members are widely distributed across mammals, invertebrates, plants, and certain fungi, playing pivotal roles in biological processes such as immune responses, pathogen recognition, and signal transduction in humans and other vertebrates. The functionality of C-type lectins is closely associated with their conserved carbohydrate recognition domains (CRDs), which adopt distinct folding structures capable of precisely recognizing and binding specific carbohydrate molecules, including mannose, glucose, galactose, and glycosaminoglycans. These glycans are critical in pathogen interactions, damaged cell recognition, and immune regulation (14, 15).

CD302, the simplest single-domain type I C-type lectin receptor within this family, integrates into cell adhesion complexes and primarily modulates cell-matrix interactions to exert essential roles in immune regulation. Dysregulation of CD302 may impair the adhesion and migratory capacities of antigen-presenting cells (APCs), disrupting their communication with the microenvironment and hindering antigen uptake, migration to lymph nodes, and subsequent T-cell activation. Such dysfunction can lead to immune abnormalities and contribute to the pathogenesis of immune-related disorders, underscoring CD302’s critical role in maintaining immune homeostasis and its potential as a therapeutic target (13). In monocytes and macrophages, CD302 facilitates multi-step immunomodulatory processes. For instance, it specifically recognizes pathogen-associated glycoproteins (21), mediates phagocytosis to clear invading microbes (22), upregulates inflammatory mediators and cytokines to coordinate innate and adaptive immunity (12), and establishes long-term immune memory through interactions with other immune cells (16).

Research on CD302 in oncology remains nascent, with heterogeneous expression and functional profiles observed across cancer types. In acute myeloid leukemia (AML), CD302 is highly expressed in bone marrow immune populations and correlates positively with CD33, a clinical AML biomarker. CD302 serves as a delivery vehicle for pyrrolobenzodiazepines (PBDs) to eliminate HL-60 leukemia cells (16). In multiple myeloma, low CD302 expression activates oncogenic MYC pathways, driving disease progression and poor prognosis, while high expression correlates with prolonged overall survival (OS) (18). In lung squamous cell carcinoma, CD302 overexpression associates with favorable outcomes, likely via suppression of tumor growth and metastasis through immunomodulation or angiogenesis regulation (20). A recent bioinformatics study further identified CD302 as a key target in the SECISBP2L-regulated angiogenesis network in LUSC, supporting its regulatory role in this subtype (23).

However, CD302’s role in other lung cancer subtypes, particularly lung adenocarcinoma (LUAD), remains underexplored. Leveraging TCGA data (59 normal and 539 tumor samples), bioinformatics analyses revealed significantly downregulated CD302 expression in LUAD tissues compared to normal counterparts. CD302 levels negatively correlated with T stage, N stage, and pathological staging but showed no association with age, sex, or M stage. Kaplan-Meier analysis demonstrated that high CD302 expression predicted improved OS and progression-free survival (PFS) in LUAD patients. Multivariate Cox regression confirmed CD302 downregulation as an independent prognostic risk factor, and a nomogram model effectively predicted 1-, 3-, and 5-year survival rates. ROC curve analysis highlighted CD302’s diagnostic potential (AUC = 0.912).

To validate these findings, 52 paired NSCLC clinical samples (tumor and adjacent tissues) were analyzed via RT-qPCR and Western blot. Results confirmed CD302 downregulation in LUAD tissues and its prognostic relevance, consistent with bioinformatics predictions. Functional assays (CCK-8, colony formation, scratch, and Transwell invasion) further demonstrated that CD302 overexpression suppressed NSCLC cell proliferation, migration, and invasion.

In summary, this study integrates bioinformatics, clinical sample analysis, and *in vitro* experiments to establish CD302 as a tumor suppressor in LUAD, where its downregulation correlates with poor prognosis and promotes malignant phenotypes. Limitations include a limited sample size, incomplete clinicopathological profiling, and unexplored signaling mechanisms. Nevertheless, these findings highlight CD302’s potential as a novel diagnostic and therapeutic biomarker in LUAD.

## 5 Conclusions

Bioinformatics analysis of public databases revealed that CD302 expression was significantly downregulated in lung adenocarcinoma (LUAD) tissues, with its levels negatively correlated with T stage, N stage, and TNM stage. Furthermore, CD302 was identified as an independent prognostic factor for LUAD patients. To validate these findings, 52 paired non-small cell lung cancer (NSCLC) samples from our institution’s thoracic surgery department were analyzed using RT-qPCR, Western blot, and clinicopathological profiling. Results confirmed marked downregulation of CD302 in LUAD tumor tissues, which strongly correlated with patient prognosis. *In vitro* functional assays demonstrated that CD302 overexpression suppressed NSCLC cell proliferation, migration, and invasion capabilities. Collectively, this study elucidates the tumor-suppressive role of CD302 in LUAD, highlighting its potential as a novel diagnostic and therapeutic marker for this malignancy.

## Data Availability

The datasets presented in this study can be found in online repositories. The names of the repository/repositories and accession number(s) can be found in the article/**Supplementary Material**.

## Supplementary material

Supplementary Figure 1 Plasmid maps of GL180 and H35378. **(A)** GL180 is an empty vector with a total length of 8604 bp. It contains an Ampicillin resistance gene (AmpR), an EF1α promoter, an EGFP reporter gene, a P2A self-cleaving peptide, a Puromycin resistance gene (Puro), a CMV promoter, a 3×FLAG tag, and a WPRE element. **(B)** H35378 is a recombinant plasmid constructed by inserting the CD302 gene into the GL180 backbone via the EcoRI and XbaI restriction sites, with a total length of 9294 bp.

Supplementary Figure 2 Melting curves of different samples in the qPCR assay. Melting curves of the GAPDH gene in H35378 **(A)**, GL180 **(B)**, and 293T **(C)**, demonstrating amplification of the reference gene. Melting curves of the CD302 gene in H35378 **(D)**, GL180 **(E)**, and 293T **(F)**, showing amplification of the target gene. Peaks in the melting curves indicate the specificity of the amplified products. A single peak suggests that the amplification product is specific and homogeneous, with no non-specific amplification.

Supplementary Figure 3 Bright field (BF) and green fluorescent protein (GFP) fluorescence microscopy images of 293T cells infected with different volumes of diluted virus (10 μL, 1 μL, and 0.1 μL).
