# Supplementary material for: CD302 regulates the malignant phenotypes of lung adenocarcinoma as a tumor suppressor gene
Source: Front Oncol. 2025 Nov 14;15:1601706. doi: 10.3389/fonc.2025.1601706 (PMC12660112; doi:10.3389/fonc.2025.1601706)
Supplement: Supplementary Figure 1 — Plasmid maps of GL180 and H35378. (A) GL180 is an empty vector with a total length of 8604 bp. It contains an Ampicillin resistance gene (AmpR), an EF1α promoter, an EGFP reporter gene, a P2A self-cleaving peptide, a Puromycin resistance gene (Puro), a CMV promoter, a 3×FLAG tag, and a WPRE element. (B) H35378 is a recombinant plasmid constructed by inserting the CD302 gene into the GL180 backbone via the EcoRI and XbaI restriction sites, with a total length of 9294 bp. [file DataSheet1.docx]

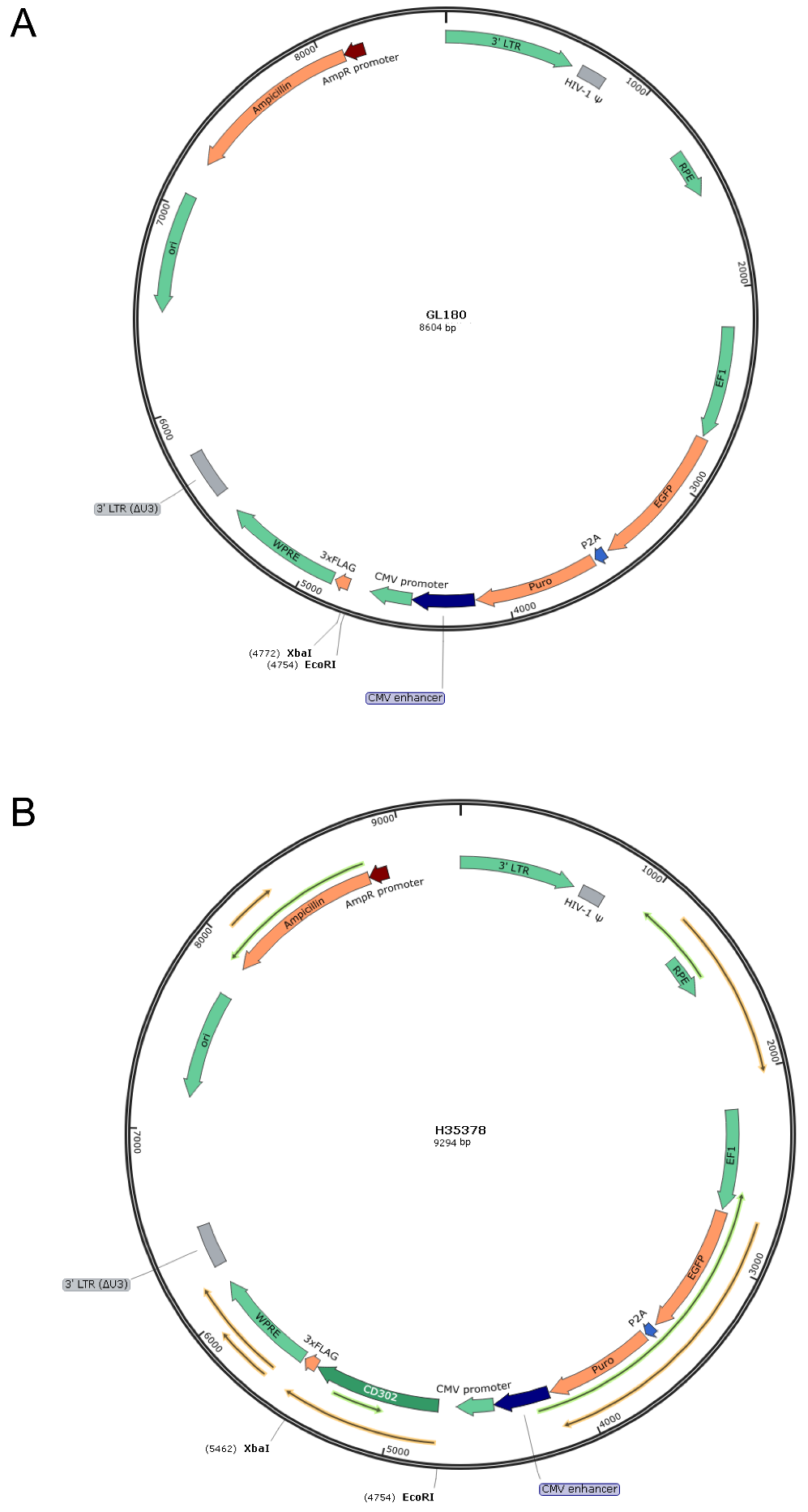


Figure S1

Plasmid maps of GL180 and H35378. (A) GL180 is an empty vector with a total length of 8604 bp. It contains an Ampicillin resistance gene (AmpR), an EF1α promoter, an EGFP reporter gene, a P2A self-cleaving peptide, a Puromycin resistance gene (Puro), a CMV promoter, a 3×FLAG tag, and a WPRE element. (B) H35378 is a recombinant plasmid constructed by inserting the CD302 gene into the GL180 backbone via the EcoRI and XbaI restriction sites, with a total length of 9294 bp.


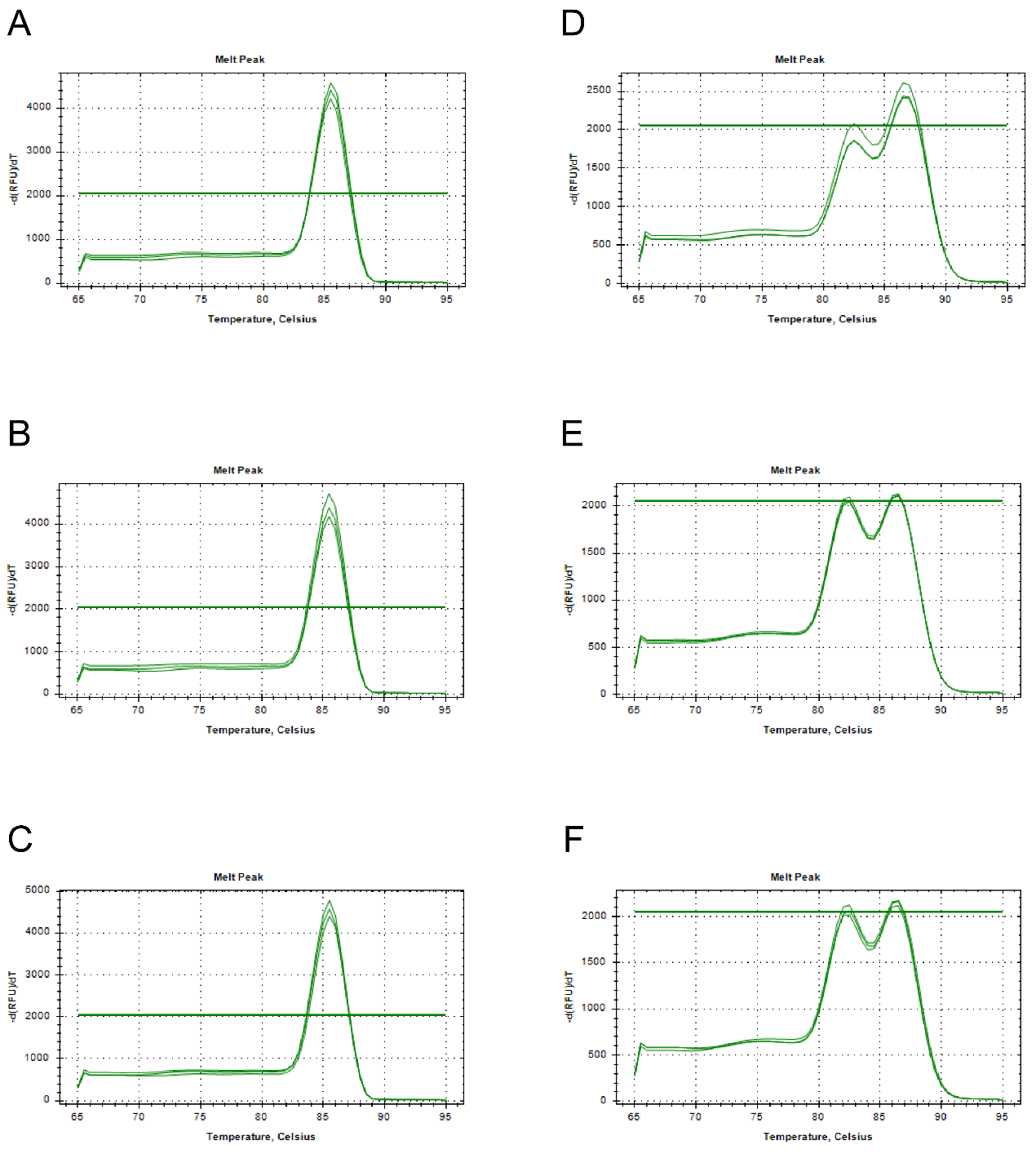


Figure S2

Melting curves of different samples in the qPCR assay. Melting curves of the GAPDH gene in H35378 (A), GL180 (B), and 293T (C), demonstrating amplification of the reference gene. Melting curves of the CD302 gene in H35378 (D), GL180 (E), and 293T (F), showing amplification of the target gene. Peaks in the melting curves indicate the specificity of the amplified products. A single peak suggests that the amplification product is specific and homogeneous, with no non-specific amplification.


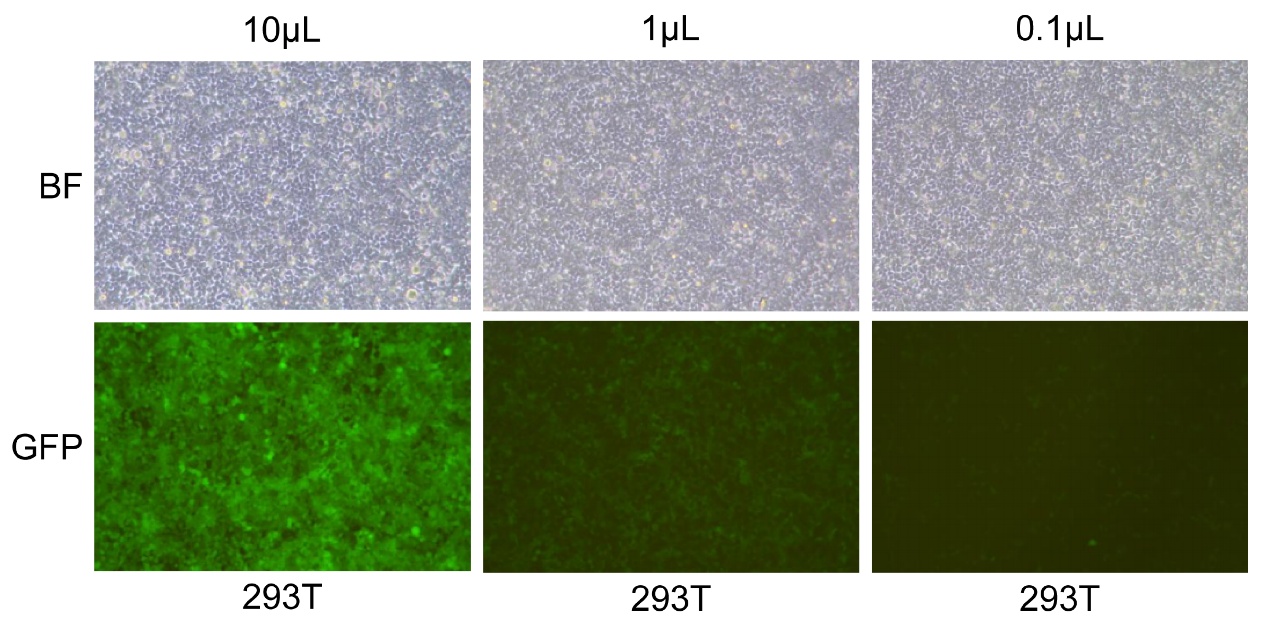


Figure S3

Bright field (BF) and green fluorescent protein (GFP) fluorescence microscopy images of 293T cells infected with different volumes of diluted virus (10 μL, 1 μL, and 0.1 μL).
