# Supplementary material for: CD302 regulates the malignant phenotypes of lung adenocarcinoma as a tumor suppressor gene
Source: Front Oncol. 2025 Nov 14;15:1601706. doi: 10.3389/fonc.2025.1601706 (PMC12660112; doi:10.3389/fonc.2025.1601706)
Supplement: Supplementary file 2 [file Table1.docx]

**Table S1** Major experimental reagents

| Reagent | Manufacturer | Country |
| --- | --- | --- |
| RPMI-1640 Medium | Gibco | USA |
| Fetal Bovine Serum (FBS) | ABW | China |
| Trypsin (1:250) | Solarbio | China |
| Cell Culture Grade DMSO | Solarbio | China |
| PBS | Biosharp | China |
| Penicillin-Streptomycin Solution | Shandong Lukang Pharmaceutical | China |
| Puromycin | Solarbio | China |
| CD302 Overexpression Lentivirus | Obio Technology | China |
| RNAiso Plus | Takara | Japan |
| PrimeScript™ RT Reagent Kit (Perfect Real Time) | TaKaRa | Japan |
| TB Green® Premix Ex Taq™ II (Tli RNaseH Plus) | TaKaRa | Japan |
| CD302 Primers | Sangon Biotech | China |
| Ethidium Bromide | Biosharp | China |
| Agarose Powder | Biofroxx | Germany |
| TAE Buffer | Biosharp | China |
| 6× DNA Loading Buffer | Biosharp | China |
| DL2000 DNA Marker | Biosharp | China |
| DEPC-treated Water | Biosharp | China |
| Absolute Ethanol | Tianjin Fuyu Fine Chemical | China |
| Chloroform | Tianjin Fuyu Fine Chemical | China |
| Isopropanol | Tianjin Fuyu Fine Chemical | China |
| 4% Paraformaldehyde | Biosharp | China |
| Crystal Violet Powder | Solarbio | China |
| CCK-8 Reagent | Abbkine | China |
| Matrigel | ABW | China |
