# Supplementary material for: CD302 regulates the malignant phenotypes of lung adenocarcinoma as a tumor suppressor gene
Source: Front Oncol. 2025 Nov 14;15:1601706. doi: 10.3389/fonc.2025.1601706 (PMC12660112; doi:10.3389/fonc.2025.1601706)
Supplement: Supplementary file 3 [file Table2.docx]

**Table S2** Construction details of the CD302 overexpression lentiviral vector

| Parameters | Value |
| --- | --- |
| Cloning Serial Number | H35378 |
| Gene Name | CD302 |
| GenBank Accession Number | NM_014880.5 |
| CDS Size (bp) | 699 |
| Species | Human |
| Cloning Restriction Sites (5' and 3') | EcoRI, XbaI |
| Prokaryotic Resistance | Amp |
| Empty Backbone Vector | GL180 pcSLenti-EF1-EGFP-P2A-Puro-CMV-MCS-3xFLAG-WPRE |
| Construct Name | pcSLenti-EF1-EGFP-P2A-Puro-CMV-CD302-3xFLAG-WPRE (H35378) |
| Sequencing Primers | Forward: CMV-F (5′-CGCAAATGGG  CGGTAGGCGTG-3′) |
|  | Reverse: WPRE-R (5′-CATAGCGTAA  AAGGAGCAACA-3′) |
