# Supplementary material for: CD302 regulates the malignant phenotypes of lung adenocarcinoma as a tumor suppressor gene
Source: Front Oncol. 2025 Nov 14;15:1601706. doi: 10.3389/fonc.2025.1601706 (PMC12660112; doi:10.3389/fonc.2025.1601706)
Supplement: Supplementary file 4 [file Table3.docx]

**Table S3** Primer information

| Gene | Primer ID | Sequence (5’→3’) |
| --- | --- | --- |
| CD302 (target) | MQ6282-1 | TCCTGCTGCCGTTGCTG |
| CD302 (target) | MQ6282-2 | CCGCTCCATGGTCAGTACAC |
| GAPDH (internal reference) | QCprimer002-F | GTCTTCACCACCATGGAGAA |
| GAPDH (internal reference) | QCprimer002-R | TAAGCAGTTGGTGGTGCAG |
