# Supplementary material for: CD302 regulates the malignant phenotypes of lung adenocarcinoma as a tumor suppressor gene
Source: Front Oncol. 2025 Nov 14;15:1601706. doi: 10.3389/fonc.2025.1601706 (PMC12660112; doi:10.3389/fonc.2025.1601706)
Supplement: Supplementary file 5 [file Table4.docx]

**Table S4** Relative mRNA expression of CD302

| Sample | GAPDH (Ct) | CD302 (Ct) | ΔCt | ΔΔCt | 2–ΔΔCt | Mean  2–ΔΔCt | Fold Change | Std. Dev. |
| --- | --- | --- | --- | --- | --- | --- | --- | --- |
| GL180 | 15.61 | 21.77 | 6.16 | -0.014 | 1.010 |  |  |  |
|  | 15.63 | 21.78 | 6.17 | -0.002 | 1.002 |  |  |  |
|  | 15.60 | 21.80 | 6.19 | 0.017 | 0.989 | 1.000 | 100% | 0.01 |
| H35378 | 15.91 | 10.42 | -5.46 | -11.64 | 3188.21 |  |  |  |
|  | 15.90 | 10.42 | -5.47 | -11.64 | 3202.29 |  |  |  |
|  | 15.85 | 10.47 | -5.42 | -11.59 | 3081.74 | 3157.42 | 315742% | 65.9 |
| 293T | 15.67 | 21.95 | 6.29 | 0.115 | 0.923 |  |  |  |
|  | 15.62 | 21.89 | 6.23 | 0.060 | 0.959 |  |  |  |
|  | 15.67 | 21.84 | 6.19 | 0.013 | 0.991 | 0.958 | 95.78% | 0.03 |
