# Supplementary material for: CD302 regulates the malignant phenotypes of lung adenocarcinoma as a tumor suppressor gene
Source: Front Oncol. 2025 Nov 14;15:1601706. doi: 10.3389/fonc.2025.1601706 (PMC12660112; doi:10.3389/fonc.2025.1601706)
Supplement: Supplementary file 6 [file Table5.docx]

**Table S5** Measurement of Lentiviral Titer

| Item | V Value | C Value | N Value | D Value | Titer | Mean Titer |
| --- | --- | --- | --- | --- | --- | --- |
| 1 | 10μL | 23.47 | 2.00E+05 | 1 | 4.69E+08 |  |
| 2 | 1μL | 1.60 | 2.00E+05 | 1 | 3.21E+08 | 4.28E+08 |
| 3 | 0.1μL | 0.25 | 2.00E+05 | 1 | 4.93E+08 |  |
