# Supplementary material for: CD302 regulates the malignant phenotypes of lung adenocarcinoma as a tumor suppressor gene
Source: Front Oncol. 2025 Nov 14;15:1601706. doi: 10.3389/fonc.2025.1601706 (PMC12660112; doi:10.3389/fonc.2025.1601706)
Supplement: Supplementary file 7 [file Table6.docx]

**Table S6** Reverse transcription reaction system

| Reagent | Amount |
| --- | --- |
| 5× PrimeScript Buffer | 4 μL |
| PrimeScript RT Enzyme Mix I | 1 μL |
| Random 6 mers (100 μM) | 1 μL |
| Total RNA | 1000 ng |
| RNase Free H₂O | Up to 20 μL |
