# Supplementary material for: CD302 regulates the malignant phenotypes of lung adenocarcinoma as a tumor suppressor gene
Source: Front Oncol. 2025 Nov 14;15:1601706. doi: 10.3389/fonc.2025.1601706 (PMC12660112; doi:10.3389/fonc.2025.1601706)
Supplement: Supplementary file 8 [file Table7.docx]

**Table S7** PCR reaction system

| Reagent | Amount |
| --- | --- |
| TB Green Premix Ex Taq II | 12.5 μL |
| Forward primer (10 μM) | 1 μL |
| Reverse primer (10 μM) | 1 μL |
| cDNA | 1 μL |
| RNase Free H₂O | 9.5 μL |
