# Supplementary material for: CD302 regulates the malignant phenotypes of lung adenocarcinoma as a tumor suppressor gene
Source: Front Oncol. 2025 Nov 14;15:1601706. doi: 10.3389/fonc.2025.1601706 (PMC12660112; doi:10.3389/fonc.2025.1601706)
Supplement: Supplementary file 9 [file Table8.docx]

**Table S8** Primer sequences

| Gene | Primer | Primer Sequence (5’→3’) |
| --- | --- | --- |
| CD302 | Forward primer | TGGAGCGGACATGATAAGCAT |
|  | Reverse primer | AGCACAGGTGTCAACTAAATCC |
| GAPDH | Forward primer | CAGGAGGCATTGCTGATGAT |
|  | Reverse primer | GAAGGCTGGGGCTCATTT |
