# Supplementary material for: CD302 regulates the malignant phenotypes of lung adenocarcinoma as a tumor suppressor gene
Source: Front Oncol. 2025 Nov 14;15:1601706. doi: 10.3389/fonc.2025.1601706 (PMC12660112; doi:10.3389/fonc.2025.1601706)
Supplement: Supplementary file 10 [file Table9.docx]

**Table S9** Composition of SDS-PAGE separating and stacking gels

| Reagent | 10% Separating Gel (10 mL) | 5% Stacking Gel (4 mL) |
| --- | --- | --- |
| Double-distilled water | 2.6 mL | 2.8 mL |
| 30% Acr-Bis（29:1） | 3.4 mL | 0.62 mL |
| 1.5 M Tris-HCl (pH 8.8) | 3.8 mL | 0 mL |
| 1.0 M Tris-HCl (pH 6.8) | 0 mL | 0.5 mL |
| 10% Ammonium Persulfate (APS) | 0.1 mL | 0.04 mL |
| 10% SDS | 0.1 mL | 0.04 mL |
| TEMED | 0.004 mL | 0.004 mL |
