# Supplementary material for: CD302 regulates the malignant phenotypes of lung adenocarcinoma as a tumor suppressor gene
Source: Front Oncol. 2025 Nov 14;15:1601706. doi: 10.3389/fonc.2025.1601706 (PMC12660112; doi:10.3389/fonc.2025.1601706)
Supplement: Supplementary file 11 [file Table10.docx]

**Table S10** Information of antibodies

| Protein | Information of antibodies |
| --- | --- |
| CD302 | Proteintech, China, 66640-1-Ig |
| Beta Actin | HUABIO, China, EM21002 |
