# Supplementary material for: CD302 regulates the malignant phenotypes of lung adenocarcinoma as a tumor suppressor gene
Source: Front Oncol. 2025 Nov 14;15:1601706. doi: 10.3389/fonc.2025.1601706 (PMC12660112; doi:10.3389/fonc.2025.1601706)
Supplement: Supplementary file 12 [file Table11.docx]

**Table S11** Baseline information of NSCLC tissue sample patient

| Number | Age | Gender | T | N | M | TNM | Differentiation | Pathological Types |
| --- | --- | --- | --- | --- | --- | --- | --- | --- |
| 1 | 76 | Female | T2b | N0 | M0 | IIA | Middle | LUAD |
| 2 | 61 | Female | T2a | N0 | M0 | IB | Middle | LUAD |
| 3 | 62 | Female | T1a | N0 | M0 | IA | Middle | LUAD |
| 4 | 76 | Male | T1c | N0 | M0 | IA3 | Poor | LUAD |
| 5 | 51 | Male | T1b | N0 | M0 | IA2 | Well | LUSC |
| 6 | 50 | Male | T1a | N0 | M0 | IA | Middle | LUAD |
| 7 | 58 | Male | T2a | N0 | M0 | IB | Poor | LUAD |
| 8 | 52 | Female | T2b | N0 | M0 | IB | Middle | LUSC |
| 9 | 67 | Male | T2a | N0 | M0 | IB | Middle | LUSC |
| 10 | 71 | Female | T1b | N0 | M0 | IA2 | Poor | LUAD |
| 11 | 58 | Female | T1b | N2 | M0 | IIIA | Poor | LUAD |
| 12 | 73 | Female | T1a | N0 | M0 | IA1 | Well | LUAD |
| 13 | 58 | Male | T2a | N0 | M0 | IA2 | Middle | LUAD |
| 14 | 41 | Male | T2b | N2 | M0 | IIIA | Middle | LUAD |
| 15 | 53 | Male | T1c | N0 | M0 | IA3 | Middle | LUAD |
| 16 | 55 | Male | T1b | N0 | M0 | IA2 | Middle | LUAD |
| 17 | 49 | Male | T1b | N0 | M0 | IA2 | Poor | LUAD |
| 18 | 58 | Female | T3 | N2 | M0 | IIIB | Middle | LUSC |
| 19 | 57 | Male | T2b | N0 | M0 | IIA | Middle | LUAD |
| 20 | 50 | Male | T2b | N1 | M0 | IIB | Middle | LUAD |
| 21 | 47 | Female | T1c | N0 | M0 | IA3 | Middle | LUAD |
| 22 | 53 | Female | T1b | N0 | M0 | IA2 | Well | LUAD |
| 23 | 67 | Male | T1b | N0 | M0 | IA2 | Middle | LUAD |
| 24 | 56 | Male | T1c | N0 | M0 | IA3 | Middle | LUAD |
| 25 | 57 | Female | T2a | N0 | M0 | IB | Poor | LUAD |
| 26 | 51 | Female | T1a | N0 | M0 | IA | Middle | LUAD |
| 27 | 70 | Female | T1a | N0 | M0 | IA3 | Middle | LUAD |
| 28 | 58 | Male | T3 | N2 | M0 | IIIB | Poor | LUAD |
| 29 | 51 | Male | T2b | N0 | M0 | IIA | Poor | LUAD |
| 30 | 76 | Male | T2a | N0 | M0 | IB | Middle | LUSC |
| 31 | 57 | Male | T2a | N2 | M0 | IIIA | Middle | LUSC |
| 32 | 70 | Male | T2a | N0 | M0 | IB | Poor | LUAD |
| 33 | 51 | Female | T1b | N0 | M0 | IA2 | Middle | LUAD |
| 34 | 49 | Female | T2a | N0 | M0 | IB | Well | LUAD |
| 35 | 51 | Male | T1b | N0 | M0 | IA2 | Poor | LUAD |
| 36 | 66 | Male | T1b | N0 | M0 | IA2 | Well | LUSC |
| 37 | 50 | Female | T1b | N0 | M0 | IA2 | Middle | LUAD |
| 38 | 59 | Male | T1b | N0 | M0 | IA2 | Well | LUAD |
| 39 | 61 | Female | T1b | N0 | M0 | IA2 | Well | LUAD |
| 40 | 63 | Male | T3 | N0 | M0 | IIB | Poor | LUSC |
| 41 | 70 | Male | T2 | N0 | M0 | IIB | Poor | LUSC |
| 42 | 49 | Female | T1b | N0 | M0 | IA2 | Well | LUAD |
| 43 | 41 | Male | T1b | N0 | M0 | IA2 | Well | LUAD |
| 44 | 69 | Male | T2a | N0 | M0 | IB | Poor | LUAD |
| 45 | 54 | Female | T2a | N0 | M0 | IB | Poor | LUAD |
| 46 | 61 | Male | T1a | N0 | M0 | IA1 | Well | LUAD |
| 47 | 54 | Female | T1c | N0 | M0 | IA3 | Well | LUAD |
| 48 | 57 | Female | T1c | N0 | M0 | IA3 | Middle | LUAD |
| 49 | 68 | Female | T2a | N0 | M0 | IB | Poor | LUAD |
| 50 | 64 | Female | T1b | N0 | M0 | IA2 | Well | LUAD |
| 51 | 20 | Male | T1b | N0 | M0 | IA2 | Well | LUAD |
| 52 | 54 | Male | T1b | N0 | M0 | IA2 | Well | LUSC |
